# An Internet-of-Things (IoT) Network System for Connected Safety and Health Monitoring Applications

**DOI:** 10.3390/s19010021

**Published:** 2018-12-21

**Authors:** Fan Wu, Taiyang Wu, Mehmet Rasit Yuce

**Affiliations:** Department of Electrical and Computer Systems Engineering, Monash University, Melbourne, VIC 3800, Australia; fan.wu@monash.edu (F.W.); taiyang.wu@monash.edu (T.W.)

**Keywords:** LPWAN, LoRa, WBAN, safety applications, wearable sensor network, Internet of Things

## Abstract

This paper presents a hybrid wearable sensor network system towards the Internet of Things (IoT) connected safety and health monitoring applications. The system is aimed at improving safety in the outdoor workplace. The proposed system consists of a wearable body area network (WBAN) to collect user data and a low-power wide-area network (LPWAN) to connect the WBAN with the Internet. The wearable sensors in the WBAN are exerted to measure the environmental conditions around the subject using a Safe Node and monitor the vital signs of the subject using a Health Node. A standalone local server (gateway), which can process the raw sensor signals, display the environmental and physiological data, and trigger an alert if any emergency circumstance is detected, is designed within the proposed network. To connect the gateway with the Internet, an IoT cloud server is implemented to provide more functionalities, such as web monitoring and mobile applications.

## 1. Introduction

The Internet of Things (IoT) has become one of the most powerful communication paradigms and attracted many research interests in the 21st century [1,2]. It can connect numerous objects, such as sensors, vehicles, houses, and appliances, together to the Internet, which allows users to share information, data and resources. The emergence of IoT has made it a key component in the environmental monitoring and health-care applications. For example, wireless sensors can be deployed in various locations to monitor environmental conditions [3], and wearable sensors can be attached to the subjects’ body to measure physiological status [2]. Those data can be transmitted to a cloud infrastructure and presented to the targeted users. However, the existing works mainly focus either on environmental or health-care monitoring applications. There is a lack of such a system which can measure both of them and provide invaluable information about environmental and physiological data at the same time.

Wearable sensor nodes are generally deployed inside a wearable body area network (WBAN) to monitor physiological signals, such as the heart rate (HR), respiration rate (RR), electrocardiography (ECG), body temperature, body position, and blood pressure (BP) [2,4,5,6]. In addition to medical applications, WBAN can also be used to monitor environmental conditions around people [7,8]. Such applications can provide useful information for users to gain a deeper understanding of their surroundings, especially for safety-related applications. For instance, in a construction site, workers’ safety and health are always a major concern in the industry.

Overexposure to solar ultraviolet (UV) radiation is a risk for public health particularly for outdoor construction workers because of the typical detrimental effects such as sun burning, long-term risk of skin cancer, and eye diseases [9]. High carbon dioxide (CO_2_) concentration may cause respiratory and headache issues [10]. Among other wearable monitoring applications, temperature and relative humidity are the most common environmental parameters that are monitored [7,8,11]. As for the physiological parameters, the heart rate and body temperature are the most commonly monitored vital signs that indicate the health status of people.

A hybrid IoT network system that can monitor real-time physiological and environmental conditions to prevent workers from being exposed to risky and hazardous situations is of great importance. If users can react to emergency or accidents in time with the access to both data at the same time, risks can be reduced.

In this paper, we present a hybrid wearable sensor network system with edge computing to improve the safe working environments and reduce the health risks in the construction industry. The proposed IoT infrastructure incorporates two networks: a WBAN for data collection using Bluetooth low energy (BLE) and an LPWAN for the Internet connection using LoRa. The environmental conditions (temperature, humidity, UV and CO_2_) and vital signs (HR and body temperature) of the subject are measured by the wearable sensors deployed in the WBAN. The data from individual sensors are transmitted using BLE within the WBAN, which will be collected and transmitted to a gateway using LoRa within the LPWAN. The gateway can act as a local server for edge computing, namely pre-processing sensor signals, displaying data and triggering alerts when emergency incurred. Finally, an IoT cloud server is designed and implemented for data storage and further functionalities, such as web monitoring and mobile applications.

When dealing with healthcare monitoring, privacy and data security should be carefully considered. Developers can help to integrate security into devices, applications, and systems [12]. For data sharing, developers can use a Client-Server model, in which the server shares a certain type of information with clients while keeping other information protected by appropriate credentials [13]. Original LoRa transmission does not require encryption; however, this can be resolved by adding data encryption to LoRa transmission. Speck by National Security Agency (NSA) is a lightweight block cipher which has been optimized for performance in software implementations and is used in our Safe Node to encrypt the wireless data transfer and improve the data security. When the users need to access the data on the server, users will be asked to enter their credentials in our web application.

The remainder of this paper is organized as follows: Section 2 discusses some related works about environmental and health monitoring in IoT applications; the overall system architecture will be described in Section 3; Section 4 illustrates the implementation of the sensor network hardware and software; Section 5 discusses the system performance and evaluation; Section 6 presents the design of the IoT gateway and the cloud service; finally, the conclusion and future works are summarized in Section 7.

## 2. Related Works

### 2.1. Wireless Technologies

Various wireless technologies have been proposed to transmit data within a WBAN, such as BLE, ZigBee, ultra-wideband (UWB), and Wi-Fi [14]. However, one single wireless technology may not be sufficient to cover both short and long range applications. BLE is an ultra-low-power and low-cost wireless protocol suitable for BAN, yet it is limited by the transmission range (within 100 m). Almost every smartphone has built-in BLE function, which can be further used as a mobile gateway [15]. ZigBee can transmit a few hundred meters, but it consumes higher power than BLE. For hospital or urban areas, Wi-Fi is usually deployed and selected for a wireless network, especially for high data rate applications. However, the power consumption is the highest among these wireless technologies.

Besides the aforementioned wireless protocols, emerging technologies such as LoRa and NB-IoT have drawn several research and industrial interests due to its low power, long range and low-cost characteristics. They are two representative technologies of low-power wide-area network (LPWAN) [16,17]. NB-IoT is a Narrow Band IoT technology that can coexist with GSM (global system for mobile communications) and LTE (long-term evolution) that operate under licensed frequency band. The coverage for NB-IoT is 18 km in urban areas and up to 25 km in suburbs [17]. It reduces the device cost and minimizes battery consumption. Although the device cost is low, the spectrum and deployment cost for NB-IoT are relatively high with >$500 million/MHz and $15,000/base station [18]. 

LoRa is a proprietary spread spectrum modulation technology developed by Semtech mainly for long-range machine to machine (M2M) and IoT applications. It is a promising solution that enables long distances connectivity at low energy budget [19] while it does not require data encryption. It can achieve up to 15 km transmission range in rural areas and 5 km in urban areas [20]. LoRa uses license-free sub-GHz frequency band for radio transmission in different areas, for example, 433 MHz and 863–870 MHz in Europe, 902–928 MHz in the US, and 915–928 MHz in Australia. LoRa is based on star network topology, where data from end devices will be transmitted to the LoRa gateway directly. In addition to LoRa, LoRaWAN includes the network layer which is built on top of the LoRa physical layer and it has the ability to send the information to any LoRaWAN gateways that are already cloud-connected. A typical LoRaWAN network architecture includes multiple sensor nodes, gateways, a network server, and application servers. The gateway dispatches the LoRaWAN frames from sensor nodes to a network server which will decode the frames, perform security checks and send the data to application servers. The application servers will receive the data from the network server and decide the action in the application [21]. In a LoRaWAN network, nodes are not associated with a specific gateway and the data from one node can be received by multiple gateways [18], which can help to reduce the overhearing of other gateways [21]. LoRaWAN provides three distinct security keys (NwkSKey, AppSKey, and AppKey) for multiple layers data security/encryption purposes [22]. However, with such network architecture, LoRaWAN can be complicated for our targeting application. There are already some existing works using LoRa/LoRaWAN in the wireless network design for different scenarios, such as environmental measurements [23], safety monitoring [7], and healthcare [24,25].

The above studies show that LoRa wireless technology has the advantages of enabling data transmission at long range and low power. Compared to NB-IoT, LoRa outweighs the NB-IoT in terms of spectrum and deployment cost. To simplify the process of building the network, LoRa is selected in our network design rather than LoRaWAN. Nevertheless, the data security issue of LoRa about wireless transmission of health data needs to be addressed. BLE can be used in the body area network for communication because of its ultra low power specifications and widely embedded in almost every smartphone, which can be used to interface with smartphone easily. Therefore, a hybrid wearable network consisting of LoRa and BLE is of great significance.

### 2.2. IoT Gateway

An IoT gateway at the edge of the network generally acts as a bridge device between the local sensor network and the cloud services [26,27,28]. Normally it receives data from the local devices and sends them directly to the cloud where data will be processed and displayed to users via web applications. Such typical usage of a gateway is not suitable for real-time applications because there will be latency between the local network and cloud. Low latency response is of great importance for some IoT projects, especially safety and healthcare situations. It is also not applicable for applications which require frequent relocation. This is because a new location may have restricted Internet access and cause difficulties when accessing the cloud service. Therefore, edge computing is proposed by researchers in the current IoT design paradigm. Edge computing is a technology allowing computation to be performed at the edge of the network rather than at the cloud [29,30]. Although fog computing focuses more on the infrastructure site, its function can be interchangeable with edge computing and can address some of those issues [29]. Edge computing has location awareness and low latency, and can support real-time interactions between the user and network [30]. The edge gateway is able to pre-process sensor data locally and send emergency notifications to users immediately without the latency from the cloud [31].

### 2.3. Monitoring Applications

With the emergence of IoT, there have been various works proposed by researchers for environmental and healthcare monitoring applications. For example, M. Chen et al. propose a smart clothing integrated with ECG, blood oxygen saturation and temperature sensors in [32]. BLE is adopted to transmit the sensor data to a smartphone which will be connected to the mobile cloud platform. The work reported in [24] presents an IoT-based health monitoring system via LoRaWAN. Physiological data such as blood pressure, glucose, and temperature from people in rural areas are transmitted to a remote LoRa server using the LoRaWAN network. As the outdoor LoRa gateway can cover large areas (33 km^2^), it can be a great alternative in places where the cellular network is not available. Several experiments have been conducted, which demonstrates that LoRa not only can monitor the health conditions of people in rural areas, but also can lower the power consumption of the wireless system compared with a traditional cellular network. In [33], a wearable ECG monitoring system is presented to connect directly with the IoT-cloud using Wi-Fi. The proposed implementation enables real-time ECG data collection, visualization, and storage, which is useful in the early diagnosis of cardiovascular diseases. Z. Zhu et al. present a systematic review of the wearable sensor systems for the health monitoring of infants in [34]. Different designs for the measurements of infants’ vital signs are introduced and compared in terms of biomedical parameters, monitoring methods, wireless techniques, and power supply.

In [11], the authors present a wearable environmental monitoring system in a large-scale urban area—Singapore. The system adopts Wi-Fi to achieve the outdoor localization rather than the Global Positioning System (GPS), because of the large size and high power consumption of GPS. Wi-Fi is also used for wireless communication, which can upload data to the cloud infrastructure based on the HTTP protocol. The proposed system can measure different environmental conditions, including temperature, humidity, light, ambient pressure, acceleration, and sound pressure. The sensor node is powered by a rechargeable lithium battery for long-term operation, which can last up to 7 days. The work in [7] presents a self-powered wearable safety monitoring application based on LoRa. The power management unit is able to harvest solar energy at its maximum power point and power the sensor nodes. The sensor node is designed to monitor safety-related environmental parameters, such as temperature, Ultraviolet (UV) index, relative humidity and carbon dioxide (CO_2_). LoRa is exerted to transmit the data from the wearable node to the IoT gateway, which will upload all the data to the cloud server for further data analysis.

As mentioned previously, most of the works are either based on environmental or physiological parameters. So far there are only a few works that can measure both aspects at the same time. For example, a smart IoT architecture system for both environmental and health monitoring is presented in [35]. The system uses an ultra-low power hybrid network consisting of two different wireless technologies: the RFID for tracking purposes and 6LowPAN for wireless sensor network (WSN). The sensor node includes a multi-sensor board that can collect both environmental data (temperature, barometric pressure, ambient light) and some physiological parameters (acceleration and ECG signals) in real-time. The data can be accessed by both local and remote users via a customized REST web service. In [36], a demonstration platform for the continuous monitoring of environmental and physiological conditions in daily life is presented. Novel flexible materials make it easily conform to the human body while measuring different parameters, such as ECG, PPG, hydration, pressure and Volatile organic compounds (VOCs).

A hybrid sensor system that can monitor both environmental and health parameters and provide on-time information to users are of significance to future safety applications. In this paper, we propose an Internet of things platform employing BLE and LoRa wireless modules to effectively facilitate a connected safety and health monitoring application. Among different wireless technologies, BLE has been widely used in BAN for a short distance and low power transmission scenarios. For long-range cases, LoRa appears to be a promising solution as it can transmit up to 15 km. It will be beneficial and achieve a good balance between power consumption and data transmission by combining two wireless technologies together.

## 3. System Architecture

**Figure 1:**
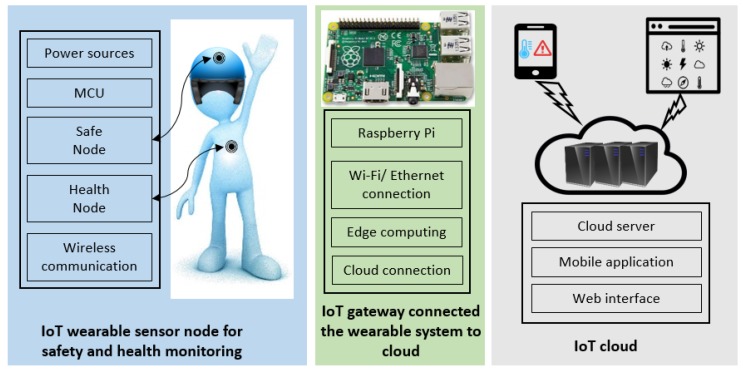
System architecture of the wearable sensor network for environmental and health monitoring.

Safety is one of the most significant considerations in an industrial workplace, where occupational injuries and illness may change the life of workers permanently. WBAN, LPWAN, and IoT infrastructures have been taken into account in our design to achieve a reliable safety monitoring system in terms of wireless technologies suitable for WBAN, network coverage range of LPWAN, sensor node’s power consumption, and the IoT cloud server. The overall system architecture comprises three subsystems as presented in Figure 1: (1) the wearable sensor nodes; (2) the IoT gateway; (3) the Internet cloud.

### 3.1. Wearable Network

There are two wearable sensor nodes on each subject: the Safe Node for environmental monitoring and the Health Node for physiological parameters’ measurements. The Health Node comprises a BLE module enabling WBAN communication, a PPG sensor for heart rate monitoring and a body temperature sensor. There are four environmental sensors on the Safe Node to measure the ambient temperature, relative humidity, CO_2_ and UV sensor. The Safe Node comprises two wireless modules: the BLE for communication within the WBAN and LoRa for transmission in the LPWAN.

The BLE in the Safe Node is responsible for receiving sensor data from the Health Node within the WBAN, which will be transmitted to a remote gateway via the LoRa network. BLE can transmit data at low power consumption and high data rate, but it is limited by the transmission range. LoRa can transmit data over a long distance while sacrificing the data rate and increasing power consumption. Therefore, in the proposed hybrid network design, LoRa is adopted for long-range data transmission and BLE is used to transmit data inside the WBAN. In addition to receiving data from the Health Node, the BLE can also transmit the wireless data to a smartphone for visualization. A web-based smartphone application is developed for this purpose.

### 3.2. IoT Gateway

The main role of the IoT gateway is to connect the wearable network to the IoT cloud and perform edge computing. The gateway consists of one Raspberry Pi, Internet connection, and a LoRa module. The Pi connected with the LoRa module receives data from the Safe Node, processes the data, and stores them into a local MySQL database. A web application that can show the data on the local website is developed for data visualization. Detailed implementations of the gateway are provided in Section 6.1.

### 3.3. IoT Cloud

The IoT cloud server receives the data from the IoT gateway and stores the data into the cloud database-MySQL. The data stored in the database can be accessed later for further analysis. A mobile application and web interface are developed as the user interface (UI). Detailed implementations of the IoT cloud server are provided in Section 6.2.

### 3.4. Network Implementation

**Figure 2:**
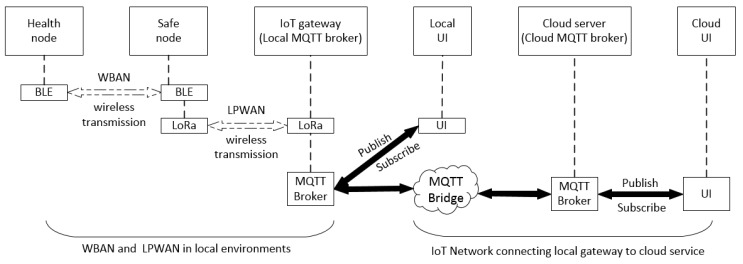
Network implementation from WBAN to the cloud server.

The proposed data flow for the entire network is shown in Figure 2. There are mainly two networks: one is in local environments—LPWAN including the WBAN, and the other one is the IoT network connecting to the cloud. As mentioned previously, the data from the Health Node and Safe Node will be transmitted to the IoT gateway and then finally to the cloud server. MQTT (message queuing telemetry transport) is used in our IoT network system to transfer the information between the gateway and the cloud server.

## 4. Implementation of the Sensor Node

MQTT is a publish-subscribe-based messaging protocol that works on top of TCP/IP protocol and requires only limited network bandwidth. It is simple and lightweight, which is ideal for IoT applications. CoAP (constrained application protocol) is an alternative to MQTT. They are both designed for resource-constrained devices [37]. However, MQTT is a many-to-many communication protocol while CoAP is based on one-to-one communication protocol for transferring state information between client and server [38]. MQTT can easily support multiple clients through a central broker and the broker can publish messages to many clients. Such a mechanism is ideal for our application because the broker needs to publish to several clients; hence, MQTT is implemented in our design.

A normal MQTT requires some clients and a broker. MQTT clients can subscribe and publish to the broker on different topics. The broker handles the client connections. A broker can be configured to work as an MQTT bridge that can connect two MQTT brokers together. In our IoT network, there is an MQTT bridge connecting two MQTT brokers together: one is the local MQTT broker installed on the Raspberry Pi and the other one is installed in the cloud server. Therefore, messages can be transferred between the local IoT gateway and the cloud server. The message/data from the local MQTT broker will be published to the local UI and the local UI subscribes to relevant topics. Similarly, the message/data from the cloud MQTT broker will be published to the cloud UI, and the cloud UI subscribes to the desired topics.

The design and implementation of Safe Nodes and Health Nodes are discussed in this section. To best use some existing hardware components, the main hardware of each board is configured from our previous projects [7,39]. Nonetheless, some major electronic components and software algorithms are re-designed according to their new requirements for this project.

**Figure 3:**
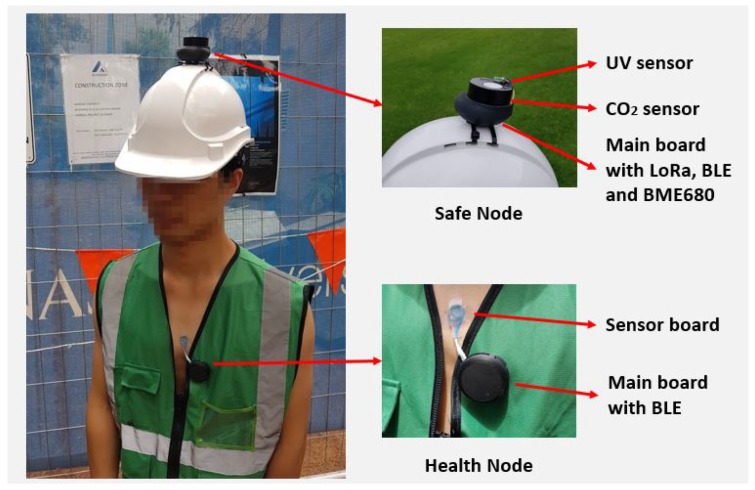
The Safe Node and Health Node are attached to the subject’ helmet and body.

Figure 3 demonstrates the wearable sensor nodes worn by the subject. The Safe Node is placed on top of the subject’s helmet, while the Health Node is attached to the subject’s chest. The Safe Node on top of the helmet is exposed to environments so that it can detect the environmental changing rapidly. The Health Node measures the body temperature and heart rate of the subject.

### 4.1. Safe Node

**Figure 4:**
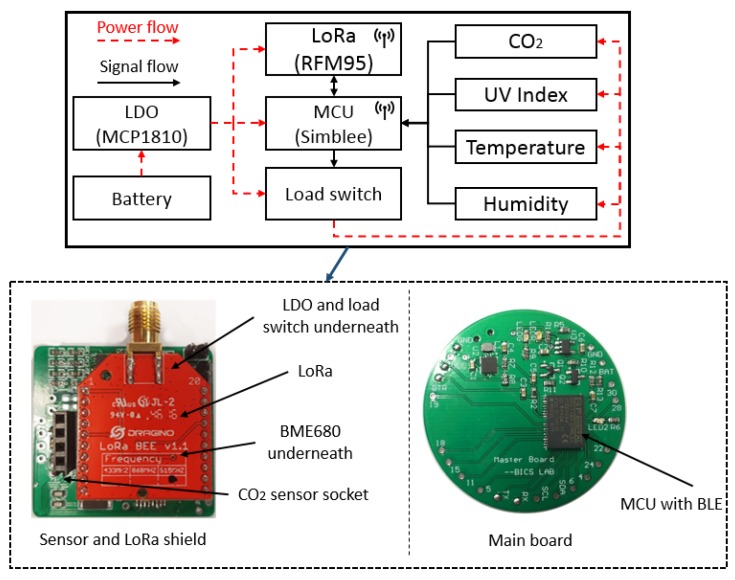
The Safe Node schematic.

The Safe Node comprises a power management unit (PMU), four environmental sensors, a microcontroller (MCU) with embedded BLE capability (Simblee), and a long-range RF module (LoRa). The sensor node from the work [7] is imported and used as the main shield for LoRa and sensors. The sensor node from the work [39] is used as the main MCU. Two boards are joint together using flexible wires by connecting the Vcc (3.3 V), Ground (GND), Inter-integrated Circuit (I2C) interface and Serial Peripheral Interface (SPI) interface. Some key electronic components used in the Safe Node are tabulated in Table 1. The schematic diagram and the figure of the Safe Node are shown in Figure 4.

#### 4.1.1. Power Management Unit

The power management unit consists of a rechargeable battery, a voltage regulator, and a load switch. The voltage regulator selected is MCP1810 from Microchips [40], which regulates the input voltage from the battery and supplies constant voltage (3.3 V) for the whole sensor node. This is an ultra-low quiescent current low-dropout (LDO) regulator that consumes only 20 nA current (typical) while delivering 150 mA current and 1 nA when the LDO is shut down. This is desirable for wearable sensor node due to its low-power consumption.

A low power, low on-resistance load switch, TPS22908 from Texas Instruments (Dallas, TX, USA), is used to turn on and off the environmental sensors at different operation stages in the design [41]. For example, the sensors can be turned off in sleep mode so as to reduce overall power consumption. The maximum quiescent and shutdown current of the switch are both 1 µA. In addition, the switch has a quick output discharge (QOD) function which will pull down the output by an internal resistor and discharge rapidly to ground level when the switch is turned off. Therefore, the devices connecting to the output pin will not be left in a floating state and cause unforeseen issues to the MCU.

#### 4.1.2. Environmental Sensors

Temperature and humidity are measured by BME680 from Bosch-Sensortech, which is an integrated environmental sensor suitable for wearable applications [42]. The sensor is a digital sensor using I2C to transfer data between BME680 and the MCU. The sensor can operate at a low voltage level (1.71–3.6 V) and consumes low current (0.15 µA) in sleep mode. The accuracy for both temperature and humidity are tabulated in Table 1.

The UV index is acquired by SI1145 from Silicon Labs [43]. This is an integrated UV index sensor with industry’s lowest power consumption (less than 500 nA in standby mode and 9 µA average in sensing mode). The sensor also has an I2C interface that is used to communicate with the MCU.

For CO_2_ measurement, a non-dispersive infrared (NDIR) sensor, COZIR-GC0012 from CO2METER (Ormond Beach, FL, USA), is selected to detect a wide range of CO_2_ concentration from 0–10,000 ppm [44]. This is a low power sensor consuming less than 1.5 mA on average. It has a short warm-up period that is less than 10 s and supports battery-operated fast monitoring applications. This is a digital output sensor which supports serial communication with MCU.

#### 4.1.3. MCU and Wireless Transmission

The Simblee RFD77101 from RF Digital Corporation (Hermosa Beach, CA, USA) is a high performance and professional grade Bluetooth Smart radio transceiver with built-in ARM Cortex M0 microcontroller. The MCU operates at 16 MHz with a 32 kHz precision crystal, 6 ADC (analog-to-digital converter) inputs, 2 I2C interfaces, and 2 SPI interfaces. The operating voltage is from 1.8–3.6 V and consumes 600 nA in ultra-low power sleep mode, 8 mA @ 0 dBm, 12 mA @ +4 dBm transmission mode, and 10 mA in receiving mode [45].

RFM95 from HOPERF Electronic is selected as the long range (LoRa) transceiver module. The RF module has high interference immunity while minimizing current consumption [46]. The transmission current is from 20 to 120 mA depending on the transmission power, while the sleep current is 0.2 µA. The RF communicates with MCU via SPI interface and some major parameters of LoRa, such as the SpreadingFactor (SF), transmission power, coding rate (CR) and bandwidth (BW), can be configured via the SPI interface.

#### 4.1.4. Software Implementation for Safe Node

**Figure 5:**
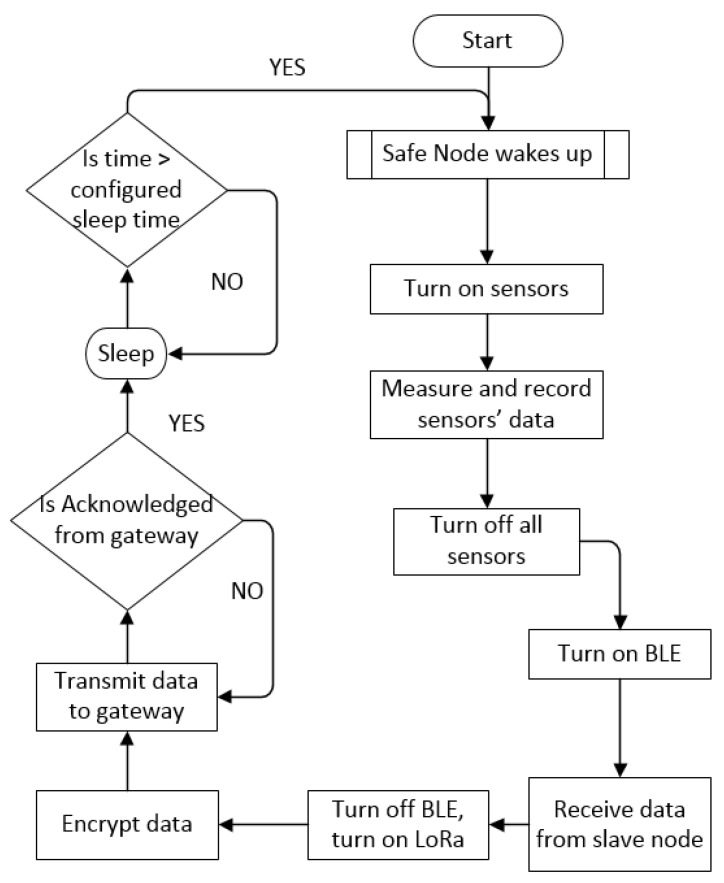
Software flowchart of the Safe Node.

Figure 5 shows the software implementation of the Safe Node. Firstly, the MCU wakes up to acquire the data from the sensors, which will be turned off after the measurements by the load switch. Then the BLE module in the Safe Node will be enabled to receive the physiological data from the Health Node within the WBAN. After the health data are collected, the BLE will be switched off while the LoRa is turned on to send data to the gateway through the LPWAN. This data is encrypted by Speck block cipher. Once the receiving acknowledge is sent back by the gateway, the Safe Node will enter the sleep cycle which can be configured according to different monitoring frequency requirements.

For data encryption, both the sender (Safe Node) and the receiver (Gateway) are using the same cipher (Speck) and the same encrypt-key. Two software libraries are used in our work including RHEncryptedDriver from RadioHead [47] and Speck from [48]. RHEncryptedDriver is a library that adds encryption and decryption to the LoRa (RFM95) driver by using the Speck cipher. At first, the RHEncryptedDriver will encrypt the LoRa data using an encrypt-key and then transmit the data to the remote LoRa gateway. After the gateway receives the encrypted data, it will decrypt the data using the same key.

### 4.2. Health Node

**Figure 6:**
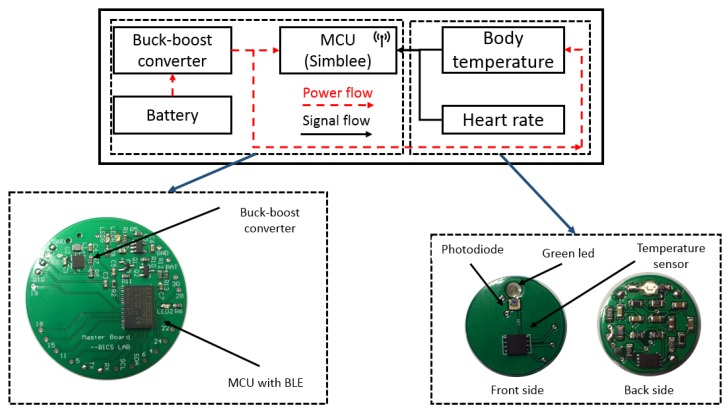
The Health node schematic.

The Health Node consists of a signal processing board for data processing and a sensor board for the measurements of HR and body temperature as shown in Figure 6. Some key electronic components used in the Health Node are tabulated in Table 2 [39].

#### 4.2.1. Signal Processing Board

The master board of the Health Node is designed for signal processing, data transmission, and power management. It uses the same MCU as the Safe Node, which will collect and process the signals from the sensor board. The calculated HR and body temperature will be sent to the Safe Node within the WBAN by BLE. All the environmental and physiological data are transmitted to the gateway of the LPWAN by LoRa, which will be stored and analyzed on the cloud.

The Health Node is powered by a 120 mAh rechargeable battery, which is regulated to a constant voltage by a buck-boost converter, RT6150A from Richtek Technology (Zhubei City, Taiwan). The RT6150A is a low-cost and highly efficient DC-DC converter with the shutdown current less than 1 µA and the quiescent current around 60 µA. The charging controller, MCP73831 from Microchip (Chandler, Arizona, USA), is adopted to manage the battery charging status to extend its lifetime. It first employs a fast charging mode with a constant current when the battery is in low capacitance and then a constant voltage mode after reaching a programmed voltage.

#### 4.2.2. Physiological Sensors

The PPG sensor is implemented with a green LED (AM2520ZGC09 from Kingbright, Taipei, Taiwan) and a surface-mounted photodiode (APDS9008 from Avago, San Jose, CA, USA). The PPG uses a green LED as it is relatively less affected by motion artifacts compared with other light [49]. The original signal from the photodiode (PD) will be pre-processed by an active low-pass filter and amplifier on the back side of the sensor board. The body temperature sensor, MAX30205 from Maxim Integrated (San Jose, CA, USA), can accurately measure temperature and provide over-temperature interrupt output to the MCU. It provides a 16-bit resolution with 0.1 °C accuracy between 37 to 39 °C. The sensor communicates with the MCU via I2C serial interface [50].

#### 4.2.3. Software Implementation

**Figure 7:**
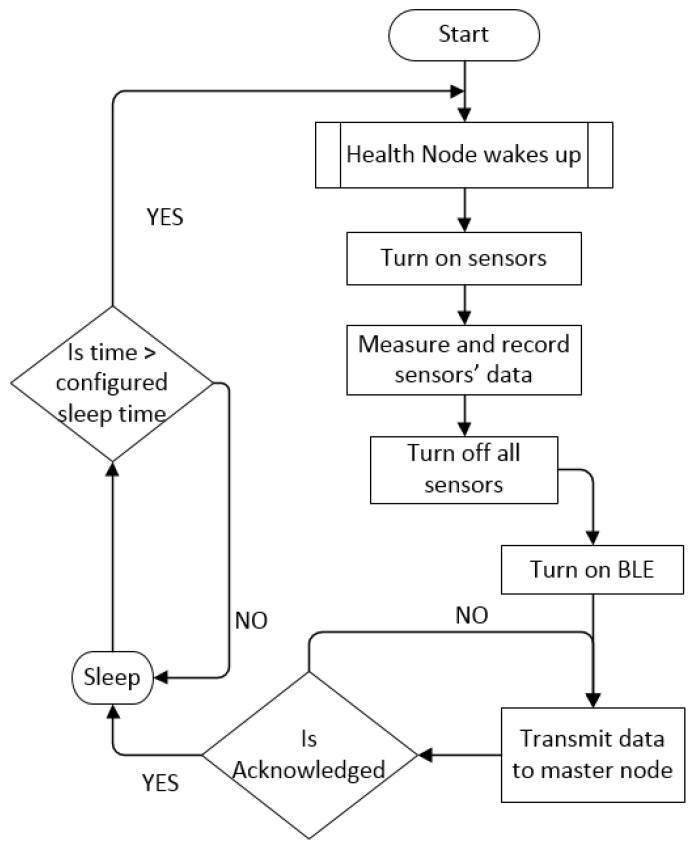
Software flowchart for the Health Node.

Figure 7 shows the software implementation for Health Node. First, the Health Node wakes up and measures the physiological sensors’ data, including body temperature and HR. After that, the BLE function of the Simblee will be enabled to transmit the physiological data to the Safe Node. Every transmission between the Health Node and Safe Node is acknowledged so that the packet success rate within BAN can be assured. Once all the data are successfully delivered, the BLE will be turned off; otherwise, a re-transmission is required. Lastly, the Health Node will enter sleep mode for the time pre-configured in the MCU software to reduce the power consumption.

## 5. System Performance Evaluation and Analysis

### 5.1. Network Coverage

To evaluate the actual LPWAN network coverage, some experimental configurations are listed below.

- Firstly, the IoT gateway is placed close to the window inside the laboratory on the second floor. Then we move the gateway to the top of the building to test the network coverage range when the gateway is at different gateway locations.
- Each sensor node is configured to send the data to the IoT gateway every minute.
- LoRa configuration: (1) Transmission power: 23 dBm; (2) Frequency: 915 MHz; (3) Spreading Factor = 128 chips/symbol (SpreadingFactor (SF) = 7); (4) BW = 125 kHz; (5) CR = 4/5.

In this work, LoRa is configured to transmit the packet at a high data rate while maintaining the long-distance transmission. Therefore, the transmission power is set to the maximum (23 dBm). With higher spreading factor, a longer transmission range can be achieved but the data rate will be lowered. Hence, to achieve high data rate transmission, the SF is set to 7. An increase in bandwidth (BW) will result in lower receiver sensitivity and higher Time on Air. BW of 125 kHz is selected as a trade-off between receiver sensitivity and Time on Air. The coding rate can be selected from 4/8, 4/7, 4/6, and 4/5. A higher coding rate will provide higher sensitivity. Therefore, CR is configured as 4/5.

**Figure 8:**
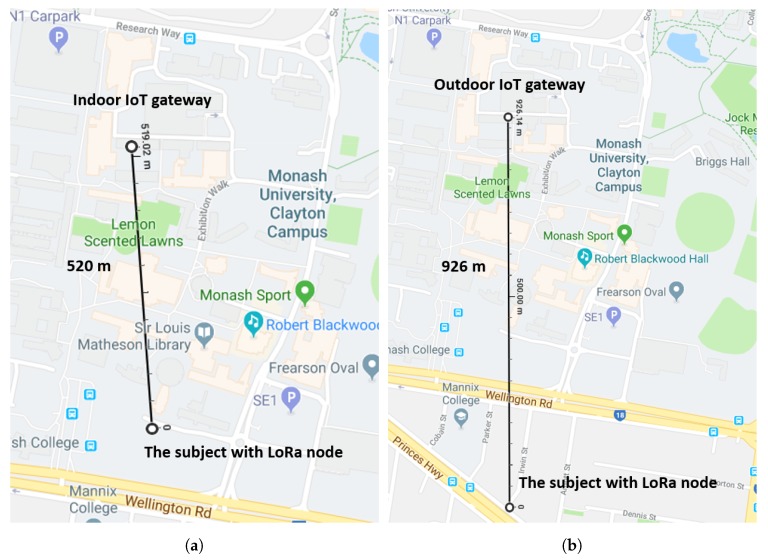
The coverage range of the IoT gateway: (**a**) indoor; (**b**) outdoor.

It can be seen from Figure 8a, when the gateway is placed indoors, the network can cover approximately 520 m. This is because there are some buildings around the testing area creating obstructions between the gateway and the sensor node. This can be comparable to a dense urban area. When the IoT gateway is moved from indoors to outdoors, the network coverage increases significantly to 926 m as illustrated in Figure 8b due to less building interference on top of the roof. Accordingly, if the gateway node is placed on top of the roof, one single gateway is able to cover the whole campus.

### 5.2. Sensors’ Performance

**Figure 9:**
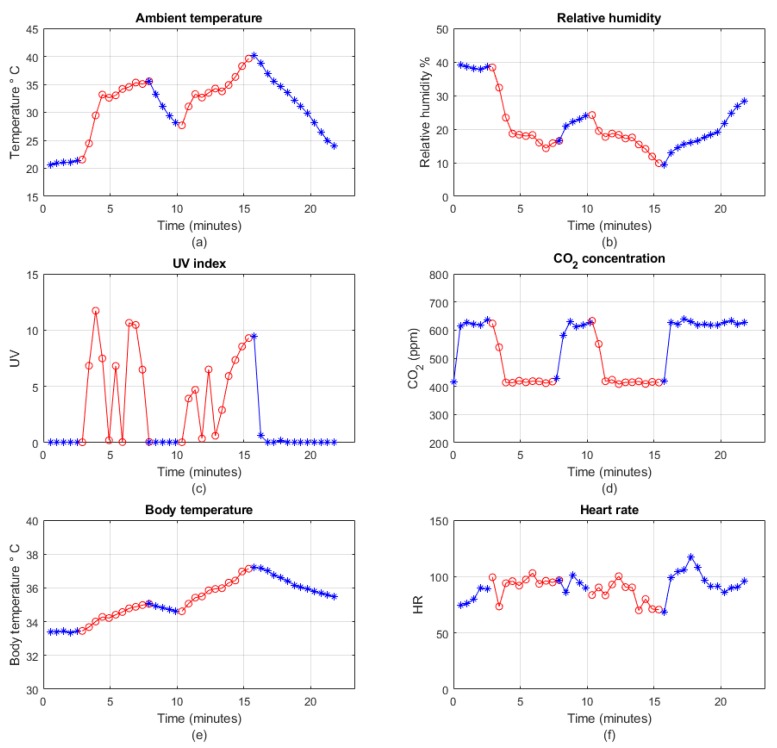
Real-time monitoring of different sensors’ data: (**a**) temperature; (**b**) relative humidity; (**c**) UV index; (**d**) carbon dioxide; (**e**) body temperature; (**f**) heart rate.

After the range test, the wearable node is worn by the subject who walks around the targeting area to check the wearable sensors’ performance in both outdoor and indoor environments. Figure 9 shows the measurements from the Health Node and Safe Node for approximately 25 min.

Figure 9a–d presents the results for ambient temperature, relative humidity, UV index and CO_2_ from the Safe Node, respectively. Figure 9e,f shows the physiological measurements for body temperature and heart rate from the Health Node. The blue lines indicate that the subject is indoor while the red lines represent outdoor conditions. It can be clearly seen that the ambient temperature and UV index are lower when the subject is indoors, but becomes higher when he is outdoors. As for relative humidity and CO_2_, they are higher when the subject is indoors and lower when the subject is outdoors. As the test is conducted in summer, the UV can be as high as 10. Some oscillations on UV measurements are observed because of the shadows from the trees and building in the area. With respect to the CO_2_ measurements, the CO_2_ concentration remains approximately 600 ppm indoors and 410 ppm outdoors. As for body temperature measurements, the temperature increases from 34 to about 37 °C and then decreases to just below 36 °C at last. This is because the body temperature increases when the subject walks outdoors. Heart rate monitoring is relatively constant in the first 15 min, after that it increases to approximately 120 because the subject is climbing the stairs at that time.

## 6. Implementation of the IoT Gateway and Cloud Server

### 6.1. IoT Gateway Implementation

#### 6.1.1. Hardware Implementation

The IoT gateway is responsible for connecting the local sensor network to the cloud infrastructure, performing edge computing inside the local network, and hosting a local server for users. Therefore, the gateway must have the following capabilities:

- Internet connection interface, such as Wi-Fi or Ethernet;
- Relatively high processing speed;
- Data storage unit, such as MySQL database;
- User-friendly interface.

In our design, the Raspberry Pi Model 3 B is selected as the main component of the gateway. The Raspberry Pi is a tiny computer with onboard Ethernet connection, Wi-Fi module, and BLE module, which makes it a suitable option for IoT applications. It supports Linux operating system called Raspbian, an open source system with great community support. The system supports many programming languages, including Python, Java, C, C++, and Node.js, etc. [51,52]. In addition, the Pi consumes very low power as compared to a normal desktop or laptop computer. The supply voltage and current are 5 V and 2.5 A respectively, which means the Pi can be powered by a portable power bank. With a 20,000 mAh portable power bank, the Pi can run without the main power supply for 8 h (20,000 mAh/2.5 A). Therefore, the gateway based on Pi can be relocated conveniently without shutting it down. The Pi also has 4 USB 2.0 ports that are able to interface with external USB devices. Although there are some other options for the gateway, such as Arduino, BeagleBone Black, Asus Tinker Board, and OrangePi Plus, Raspberry Pi is selected in our design. It is because of the huge community support, low power consumption, flexible operating system, many supported programming languages, and costs. The functions of the Raspberry Pi as a gateway are summarized here:

- One USB port of Pi is used to connect the LoRa, which enables the data acquisition from the local sensor network to the gateway database.
- MySQL database is installed in the Raspbian system for data storage. The data can be accessed in future if required.
- The built-in Wi-Fi module is used for Internet connection.
- MQTT messaging protocol is installed in the system for transmitting the data to the cloud server.
- A light-weight web server based on Node.js is installed and can be accessed via a smartphone and web browsers.

#### 6.1.2. Software Implementation

**Figure 10:**
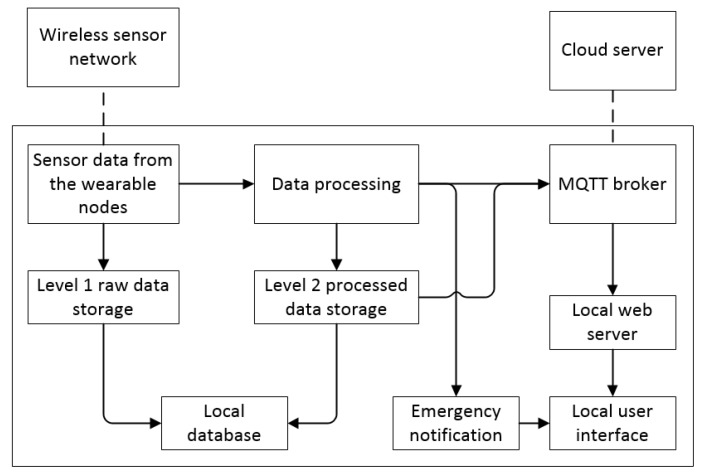
The software flowchart of the IoT gateway.

The software algorithm of the IoT gateway is illustrated in Figure 10. At first, the sensor data is received from the sensor nodes through the WBAN and LPWAN. The raw data is stored into the MySQL database without processing, which is known as level 1 data storage. The level 1 raw data is mainly for backup purpose and can be retrieved in the future for further analysis. The Pi will also conduct preliminary data processing, for example, filtering the data, detecting emergency conditions, and storing the filtered data into level 2 data storage. The data will be transmitted to the cloud via the MQTT bridge. A local web server is subscribed to different topics and display the sensor network’s data in the website. If an emergency condition is detected, it will notify the user via the website.

**Figure 11:**
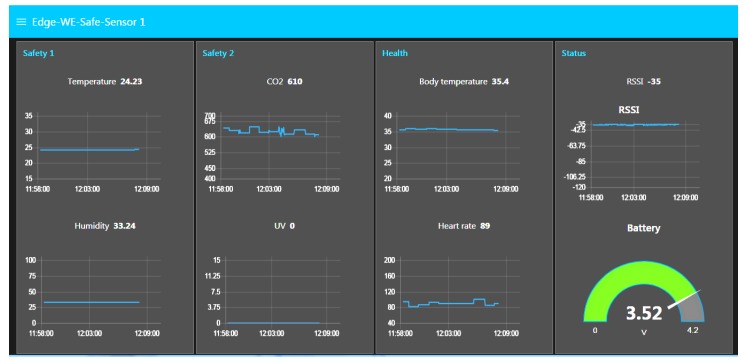
The website on the local IoT gateway.

A screen-shot of the website from the local web server is shown in Figure 11. As can be seen from the figure, the 10 min of environmental and physiological data from the Safe Node and Health Node are displayed on the website. During the period, the subject remains in the indoor environment and is close to the gateway. Therefore, both environmental and physiological data remain stable. The sensor node’s status, which includes received signal strength indicator (RSSI) and battery level, is also shown on the website.

**Figure 12:**
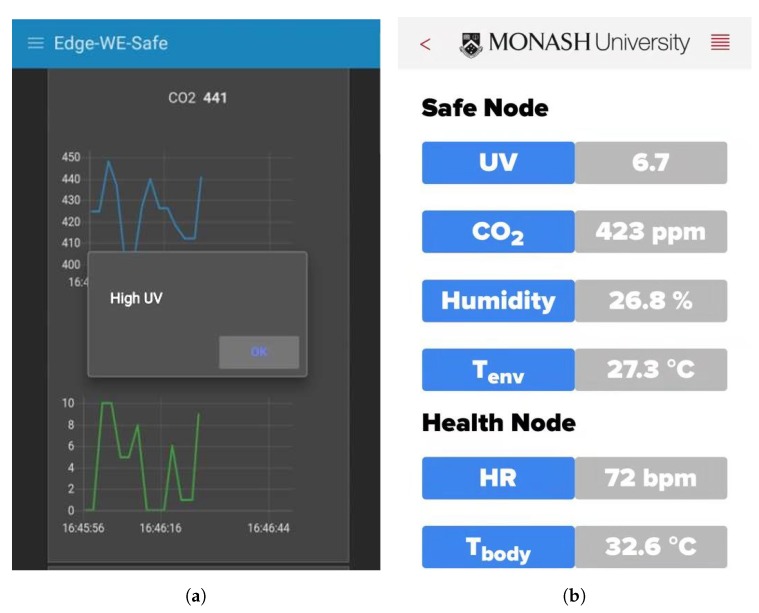
The mobile website hosted on the IoT gateway and the mobile application. (**a**) A screen-shot of the mobile website. (**b**) A screen-shot of the mobile application.

A screen-shot of the website from the mobile phone is shown in Figure 12a. As can be seen from the figure, when high UV is detected, it will notify the user by pushing notifications to the mobile phone so that the user will take some appropriate actions as presented in Table 3.

In addition to the website application for sensor data display and notifications, a web-based smartphone application for receiving wireless data directly from the Safe Node BLE function is shown in Figure 12b. The mobile application is developed using the Evothings^®^ platform. Evothings^®^ is a platform for developing a mobile application for IoT, which is coded in HTML, CSS, and Javascript.

### 6.2. IoT Cloud Server

The project’s cloud server is deployed in DigitalOcean, which is an American cloud infrastructure provider. The cloud server runs on Ubuntu 16.04.5 with 2 GB memory and 25 GB disk, which is sufficient for our system. MQTT bridge is installed on the Ubuntu server to receive the data from the IoT gateway. Once the data is received from the gateway, they will be inserted into the cloud MySQL database. The web application will query the data from the server and display to relevant users through the website.

**Figure 13:**
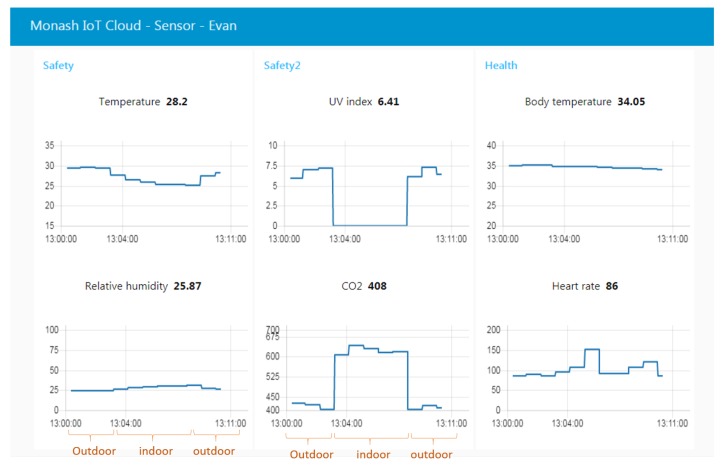
Website located on the IoT cloud.

Figure 13 presents a screen-shot of the website based on the cloud server. The figure shows 10 min continuous monitoring of real-time environmental and physiological data from a wearable sensor node worn by the subject Evan. Evan first stays outdoors then moves to indoors, and finally goes outdoors. It can be seen that when the subject is indoors, the UV and temperature detected by sensors are lower than those outdoors. However, when he stays outdoors, the CO_2_ and relativity humidity are higher than he stays indoors, and vice versa.

## 7. Conclusions and Future Works

This paper presents the implementation of a hybrid wearable sensor network system for an IoT-based industrial safety monitoring applications. It comprises a WBAN for short-range wireless communication and an LPWAN for long-distance data transmission. Two sensor nodes, the Safe Node and Health Node, are deployed in the WBAN to collect the environmental and physiological data of the subject respectively, which will be further sent to an IoT gateway via the LPWAN infrastructure. The gateway (local server) is configured to perform the edge computing function, including receiving sensor signals, processing raw data, real-time display, emergency notification, as well as sending data to the Internet cloud server. The cloud will provide the IoT applications of the system, such as data storage, website display, and mobile user interface. The proposed IoT enabled wearable sensor system can be used in the industrial safety monitoring applications, such as the construction workplace, where both the environmental condition and the health status of the workers are important to ensure safety. Table 4 summaries some recent wearable environmental and physiological monitoring applications. 

In our future work, a smart IoT gateway that can cope with multiple wireless technologies and perform faster edge computing will be deployed. The edge computing can reduce the latency and improve the efficiency of the network system. A robust smartphone-based gateway can be further developed to reduce the dependency on the Raspberry Pi and best use the BLE function of the smartphone. Since the data security is very important for personal health data, security algorithm on both edge computing and cloud services will be developed to improve the privacy and security level of the entire system. More physiological parameters and different safety-related environmental sensors can also be integrated into the current work to provide a diverse monitoring system. In addition, more subjects will be involved to validate and improve the reliability and accuracy of the IoT network system.

## Figures and Tables

**Table 1:** Key components used in the Safe Node.

Parameters	Model	Main Specifications	Power
MCU	Simblee	32-bit ARM Cortex-M0 16 MHz, 29 GPIOs	Operating voltage: 1.8–3.6 V <600 nA in sleep mode
BLE	Simblee	−93 dBm receiver sensitivity	8 mA TX @ 0 dBm 10 mA Rx
LoRa	RFM95	−148 dBm receiver sensitivity	20–120 mA TX 10 mA RX, 0.2 µA Sleep
LDO	MCP1810	Input voltage: 3.6–5.5 V	20 nA quiescent current 1 nA @ sleep
Switch	TPS22098	Input voltage: 1–3.6 V	1 µA quiescent current 1 µA @ sleep
Temperature	BME680	−40–+85 °C	0.15 µA @sleep 2.1 µA measuring
Relative humidity	BME680	0–100% RH	same as above
CO_2_	COZIR-GC0012	0–10,000 ppm	1.5 mA @ 3.3 V
UV	SI1145	1–11+ Index	500 nA Sleep, 9 µA average

**Table 2:** Key components used in the Health Node.

Parameters	Model	Main Specifications	Power
MCU	Simblee	32-bit ARM Cortex-M0 16 MHz, 29 GPIOs	Operating voltage: 1.8–3.6 V <600 nA in sleep mode
BLE	Simblee	−93 dBm receiver sensitivity	8 mA TX @ 0 dBm 10 mA Rx
Buck-boost converter	RT6150A/B	Input & output voltage: 1.8–5.5 V	<1 µA shutdown current
Charging controller	MCP73831	fast charging mode constant voltage charging mode	Charging current: 15 mA–500 mA
Body temperature	MAX30205	0.1 °C (37 to 39 °C)	Operating voltage: 2.7–3.3 V Supply current: 600 µA
PPG	LED: AM2520ZGC09 PD: APDS9008	Peak wavelength: 525 nm Peak sensitive wavelength: 565 nm	LED voltage: 1.6–5.5 V PD supply current: 42 µA

**Table 3:** Examples of safety alert using different sensors’ data.

Sensor	Data	Alerts and Action
Temperature	>30	remind the subject to rest and drink more water
UV	>5	remind the subject to rest and avoid working under direct sunlight
CO_2_	>800	notify the subject to avoid working for too long in a poor air condition environment
Heart rate	>140	notify subject to rest
Body temperature	>35	notify subject to rest

**Table 4:** Comparisons of wearable environmental and physiological monitoring applications.

Parameters	[32]	[35]	[36]	[53]	This Work
MCU	-	Cortex-M3	CC2540	CC2541	Cortex-M0
Wireless technologies	BLE	6LoWPAN RFID	BLE	BLE	BLE and LoRa
Range	Short	Short	Short	Short	Short to Long
Physiological parameters	ECG, respiration, heart rate, body temperature, blood oxygen	Motion, ECG	PPG, hydration	PPG, motion, Skin impedance, ECG, VOC, ozone, respiratory rate	Body temperature, heart rate
Environmental parameters	temperature, humidity, noise, air quality	Temperature, barometric pressure, ambient light	pressure, gas, VOC	Ambient temperature, relative humidity	Ambient temperature, relative humidity, UV, CO_2_
IoT realization	Yes	Yes	-	-	Yes
Sensor node location	Cloth	-	Wrist	Chest, wrist, handhold	Top of helmet, chest
Power requirements	Rechargeable battery	3-V rechargeable battery	Solar with 20 mAh rechargeable battery	Rechargeable battery	3.6-V rechargeable battery
Application	Healthcare	Healthcare system for hospital	Healthcare	Healthcare for Chronic Respiratory Disease	Safety and health monitoring for industrial workplace
